# Conservative management of mandibular fractures in pediatric patients during the growing phase with splint fiber and ligature arch wire

**DOI:** 10.1186/s12903-023-03309-z

**Published:** 2023-08-28

**Authors:** Lifeng Li, Kiran Acharya, Bedana Ghimire, Yanqiu Li, Xiaotao Xing, Xiaoru Hou, Lingnan Hou, Xiaoyi Hu

**Affiliations:** 1 Key laboratory of Shaanxi Province for Craniofacial Precision Medicine Research, College of Stomatology, Xi’an Jiaotong University, Xi’an, China; 2 Clinical Research Center of Shaanxi Province for Dental and Maxillofacial Diseases, College of Stomatology, Xi’an Jiaotong University, Xi’an, China; 3 Department of Cranio-Maxillofacial Trauma and Plastic Surgery, College of Stomatology, Xi’an Jiaotong University, Xi’an, China; 4 Shree Birendera Sainik (Army Hospital), Kathmandu, Nepal; 5 Department of Cranio-Maxillofacial Trauma and Plastic Surgery, College of Stomatology, Xi’an Jiaotong University, No 98 Xiwu Road, 710004 Xi’an, Shaanxi People’s Republic of China

**Keywords:** Maxillofacial trauma, Mandible fracture, Condyle, Ramus, Quartz splint

## Abstract

**Purpose:**

The purpose of this article is to discuss the effective management of mandibular fractures in pediatric patients during the growing phase of the mandible using splint fiber and ligature wire.

**Methods:**

A retrospective study examined pediatric patients with mandibular fractures who were treated using the splint (Quartz) fiber and ligature wire technique at the Stomatology Hospital of Xi’an Jiaotong University from August 2021 to January 2023. Data on gender, age, location or site of the fracture, and development of tooth stage were collected from the patient’s medical records. Descriptive statistics were used to analyze the data and evaluate the effectiveness of the splint (Quartz) fiber technique for treating mandibular fractures in pediatric patients.

**Results:**

Out of 256 subjects, 6 pediatric patients with mandibular fractures were selected, resulting in an incidence rate of 2.34% with an equal sex ratio. Mental or symphysis fracture was the most common site for fracture in children, accounting for 100% of cases. Right mandibular angle fracture was observed in 16.7% of patients, while 50% of the group (3 individuals) suffered from left condylar fracture and 16.7% had a bilateral condylar fracture. Treatment with Quartz splint fiber and circumdental arch wiring using ligature wire was successful with no observed post-treatment complications or malocclusion. The splint fiber was worn for 30 days and the circumdental arch wiring was for the same. Healing of bone fracture yields good results after 12 weeks. Follow-up care is crucial to monitor for complications, in this study, no post-treatment complications were observed.

**Conclusion:**

The treatment of pediatric mandibular fractures is complex and requires careful consideration of various factors. Conservative management should be the first choice, with open reduction and internal fixation reserved for specific cases. The use of quartz splint fiber and ligature wire is an effective treatment option for stabilizing the mandible and providing occlusal stability in growing children. A fiber splint along with ligature wire can also be used as an alternative treatment to avoid any adverse effects on the growth and development of the mandible and permanent teeth. A multidisciplinary approach is essential to achieving the best outcomes for pediatric patients with mandibular fractures.

## Introduction

Pediatric mandibular fractures occur at a relatively low frequency compared to adults, accounting for approximately 5% of all maxillofacial traumas [1]. The incidence is occurrence of facial fractures is exceedingly uncommon in children under the age of 5 years. It is approximated that merely 1% of facial fractures transpire in toddlers and preschool-aged maxillofacial trauma [2]. In children, mandible fractures are the second most common type of facial fractures, with the condyle and symphysis being the most frequent sites. Symphysis and para-symphysis fractures are more common due to developing canine tooth buds that can create a stress point in this location. Condyle and symphysis fractures account for a significant proportion of cases in children, while body, angle, and ramus fractures are relatively low in incidence but increase with adolescence. Dentoalveolar fractures are also common but are often treated in an office setting and not reported [3]. Mandibular fractures in children are more prevalent in boys and tend to occur in patients older than 2 years, particularly involving the chin or mental area [4]. Facial fractures are more common in males than females and increase with age in children. Children aged 6–11 years have a high incidence of facial fractures, with motor-vehicle accidents, play, and bike riding as common causes. Adolescents aged 12–18 years have the highest incidence, with violence and sports-related injuries as leading causes. Mandibular fractures are associated with falls, road traffic accidents, and child abuse. Sports injuries contribute to 20–30% of all oral and maxillofacial traumas in active adolescents [5–7].

The mandibular bone in children has thin cortical bone, a thick layer of adipose tissue, and a relatively larger amount of medullary bone, making it highly elastic and prone to greenstick fractures [8].

Managing mandible fractures in pediatric patients with primary or mixed dentition is challenging as treatment must consider future growth and development. Minimizing morbidity is a priority, and conservative treatment options are preferred. Internal fixation should only be considered when necessary. Treatment techniques providing a return of pre-injury occlusion and short-term immobilization with circumdental arch wiring can yield good outcomes. Age-based categorization reveals distinct patterns of injury in children with facial fractures [7, 9, 10].

Unlike the treatment principles for mandibular fractures in adults, which typically involve reduction, fixation, and immobilization, managing mandibular fractures in children requires a more cautious approach to avoid adverse effects on skeletal growth and dental development [11, 12]. Tailored treatment plans considering age, anatomy, and fracture location are necessary, and conservative methods like splinting for close reduction and stabilization are often employed [13–15]. Diagnosis of mandibular fractures in pediatric patients involves imaging techniques like orthopantomogram (OPG), Towne’s view, and 3D cone-beam computed tomography (CBCT) [16].

The growth of the mandible in children involves a gradual reduction in the cranium-to-face ratio, chin prominence development, and eruption of primary and permanent teeth [17].

This article focuses on the conservative management of mandibular fractures in pediatric patients during the growing phase using splint fiber as a treatment approach.

## Information and methodology

### Criteria for patient selection

This retrospective study examined the period from August 2021 to January 2023 and focused on patients who had been treated in the Maxillofacial Department of Trauma and Plastic Surgery Unit at the Stomatology Hospital of Xi’an Jiaotong University. A total of 256 patients were included in the study, with 6 of them being pediatric patients who had been diagnosed with mandibular fractures in various locations.

This study was conducted under the relevant guidelines and regulations approved by the Clinical Research Center of Shaanxi Province for Dental and Maxillofacial Diseases, College of Stomatology, Xi’an Jiaotong University. Ethical approval for the research was obtained from the ethical committee of the Stomatology Hospital of Xian Jiaotong University. It was confirmed that written and informed consent was obtained from all the subjects and/or their legal guardians before the start of the study.

The medical records of the patients were analyzed to obtain data on their age, gender, type, and location of mandibular fracture. The collected data were then analyzed using appropriate statistical methods descriptive analysis were calculated to determine the prevalence and characteristics of mandibular fractures in pediatric patients treated at the hospital during the study period. Descriptive analysis allows us to summarize and present the data in a concise and informative manner. By calculating the percentage of children with each type of fracture, we can identify the most common fracture types in the pediatric population.

### Materials

**Fig. 1:**
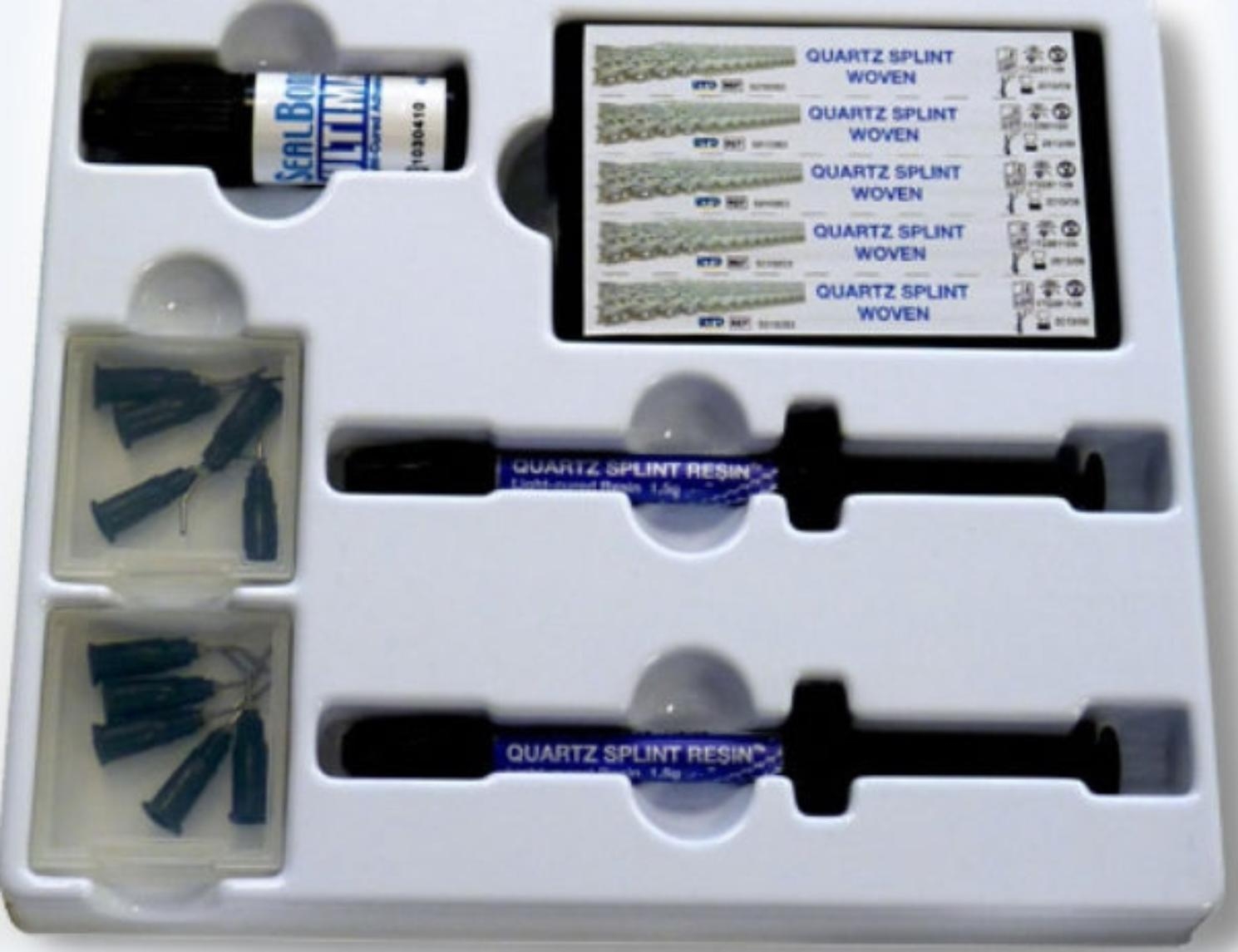
Quartz splint with full etching–bonding system

Teeth were stabilized using Quartz Splint Woven mesh quartz fiber strips and supporting high-flow resin, with a 3 M full-etching bonding system as shown in Fig. 1. Other materials included a polishing brush, polishing paste, bite paper, diamond bur, dental floss, scissors, periodontal probes, occlusion papers, and 25 mm ligature wire to stabilize the mandibular arch.

### Methods

First and foremost, patients undergo a clinical examination to assess any discrepancies or changes in occlusion. This includes palpating the affected area to identify any bony steps or irregularities. Additionally, patients may be recommended to undergo radiographic imaging to obtain further information about the fracture site. Pediatric patients are treated under general anesthesia due to their uncooperative nature. The surgical protocol for general anesthesia is followed, and the patient is placed in a supine position. Antiseptic is used to disinfect the surface area in the head, neck, chest, and mouth after the effect of general anesthesia. The teeth designated for fixation are cleaned and polished.

**Fig. 2:**
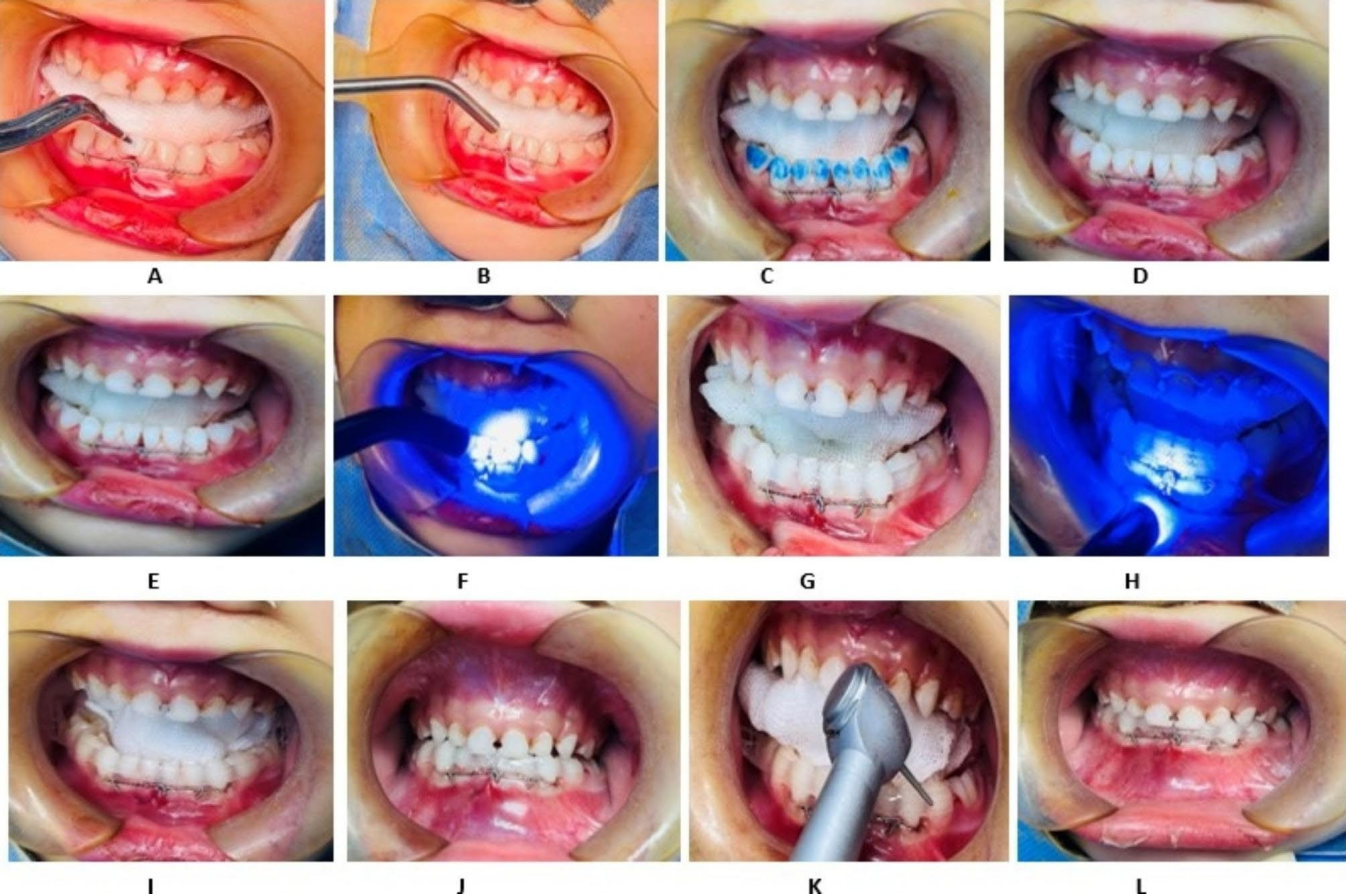
**A**- **L** showing the steps of fixation of quartz splint and circumdental wiring on pediatric fracture mandible. **A.** pre-operative stage, **B.** isolation of operative site, **C.** Acid-etching on the tooth surface, **D.** After Acid etched –tooth is washed with water and dried, **E. **Bonding agent is applied as a adhesive, **F.** light curing after bonding agent, **G.** Placement of quartz split along with flowable resin, **H.** Curing quartz splint and resin material with light-cured, **I.** adjustment of splint fiber, **J. **checking the occlusion, **k.** final adjustment of occlusion reducing the high bite points, **L.** final step with fiber splint and stable occlusion

Manual reduction is done using ligature wire for circumdental wiring for the fracture site and occlusion is adjusted. The teeth designated for fixation are cleaned, dried, and polished. Acid etching is done on the middle third of 3–4 teeth from both sides of the fracture for 45 s. A bonding agent is applied, and high-flow resin is delivered uniformly in the planned fixation area. Quartz fiber strips are wrapped with high-flow resin and shaped to fit perfectly with the tooth surface. High-flow resin is applied again on the surface of the fiber strip under the isolation of a small shield of the light-curing machine providing a tight fixation. This process is repeated for each tooth for a complete fit and fixation. Finally, occlusions are rechecked, and a final polishing is performed, as shown in Fig. 2. Radiographic examinations were conducted during the follow-up period for pediatric mandibular fractures. Follow-up appointments were scheduled at 1, 3, 6, and 12 months. If the arch showed stabilization within the first month, a cone-beam computed tomography (CBCT) scan was performed. However, if the arch did not achieve stability, the CBCT scan was planned for the next interval period.

## Results

In a group of 6 pediatric patients under pediatric care, a striking gender balance was observed, with an equal distribution of 3 Males patients (50%) and 3 Female patients (50%). The age range within this group was quite broad, with the youngest being a mere 1-year-old, and the eldest being 8 years of age. However, the average within each group was found to be distinct, with male patients averaging at 2 years old, and female patients at 5.3 years old.

All 6 patients suffered from the distressing condition of mental fracture. In addition, 1 patient exhibited a Right mandibular angle fracture, which represents 16.7% of the group’s overall condition. Half of the patients in the group, or 3 individuals (50%), suffered from left condylar fracture. This is a particularly noteworthy observation, as this type of fracture typically only accounts for a small percentage of cases. Furthermore, 1 patient was found to have a bilateral condylar fracture, making up an additional 16.7% of the group’s conditions. Data is shown in Table 1.

**Table 1:** showing the distribution of fracture

S. No.	Age of patients In Years	Gender	Location of fracture	Fixation method	A time period of fixation	Complication
1	2	Male	*Mental*	Splint	30 Days	*No*
2	1	*Male*	*Mental & Left Angle*	Splint	30 Days	*No*
3	5	*Female*	*Mental & Left Condylar*	Splint	30 Days	*No*
4	8	*Female*	*Mental & Left Condylar*	Splint	30 Days	*No*
5	3	*Male*	*Mental & Bilateral Condylar*	Splint	30 Days	*No*
6	3	Female	*Mental & Left Condylar*	Splint	30 Days	*No*

**Fig. 3:**
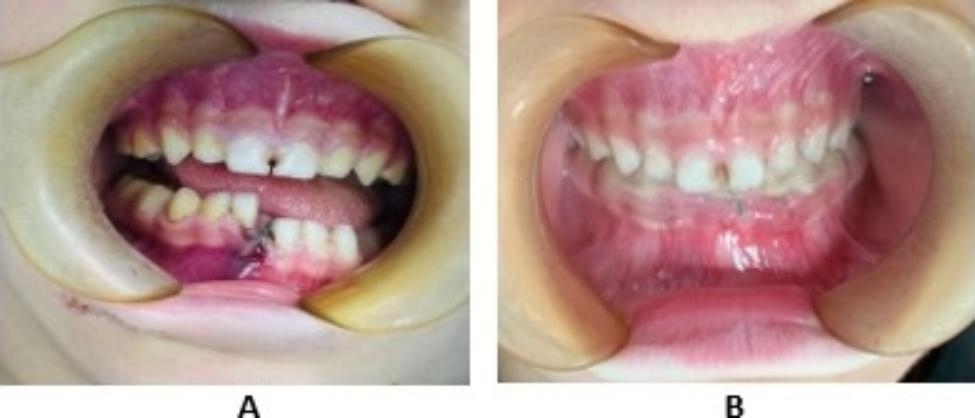
Photographs showing (**A**) Before surgery (**B**) After surgery 1 month follow –up during period of removable of splint

**Fig. 4:**
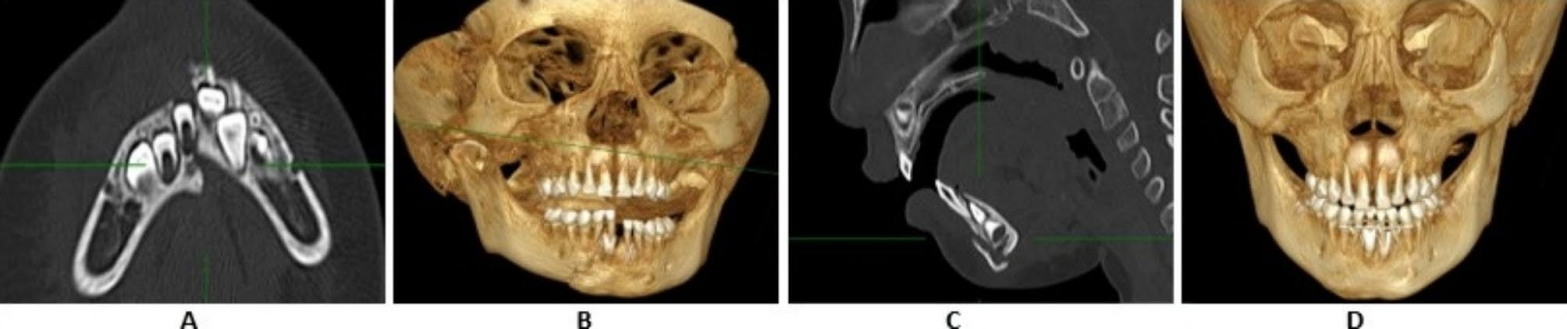
3-D CBCT showing **A**-**C**. Patient having mental fracture with obvious dislocation **D**. patient having mental fracture pre-operative after 6 months

After the completion of a 30-day observation period, which included circumdental arch wiring, the ligature wire and quartz splint was removed. The effectiveness of the 4 weeks quartz splint fixation was found to be 100% based on the results obtained from the CT scans conducted on all patients. The results showed that the desired outcome had been achieved, as shown in Figs. 3 and 4. No, the complication was noticed.

- ***Patient was recalled for follow up in 1 months however, due to the Covid-19 pandemic, the patient was unable to visit within the intended timeframe. Despite the delay, the healing of the bone was consistently observed to be excellent through CBCT evaluations.***

## Discussion

Mandibular fractures are relatively rare in pediatric patients, but they are the most common facial injury in this age group. The unique anatomical and physiological characteristics of pediatric patients, such as softer bone structure and developing dentition, make the management of mandibular fractures different from that in adults [18]. The management of mandibular fractures in children differs significantly from that in adults due to the potential disruption of growth. In pediatric patients, the outcome of mandibular fracture treatment is not solely determined by the initial intervention, but also by the impact of growth on both form and function. This highlights the importance of carefully considering the long-term effects of any treatment plan on a child’s growing facial structures, to promote optimal healing and prevent any potential complications that may arise later in life [19]. Due to the shape and size of deciduous crowns in pediatric patients, the placement of circumdental wires and arch bars during open reduction and fixation may be more challenging. Additionally, the presence of tooth buds within the mandible must be taken into account during surgical procedures, as any trauma to these developing buds may impede the eruption of permanent teeth and potentially result in a narrow alveolar ridge. Therefore, special care must be taken during the management of mandibular fractures in children to minimize the risk of damaging developing dentition and promote proper growth and development of the oral structures [20]. For growing mandible, the quartz splint helps the mandibular arch to stabilize, the maxillary arch is kept as usual and the patient is advised to have a soft liquid diet and maintain good oral hygiene. These splints are more reliable than open reduction or intermaxillary fixation techniques with cost-effectiveness the patient which saves the patient time, pain, and discomfort. On a different Literature review suggest that using impression material, impression was taken for fabrication of splint and custom splint were used for fracture [21]. On our research we use prefabricated quartz splint fiber with resin cements.

Quartz splints have been found to be reliable in stabilizing the mandibular arch, providing advantages such as cost-effectiveness, reduced surgical trauma, and lower risk of complications.

Follow-up appointments are scheduled at regular intervals to monitor the healing progress. No complications were observed during the follow-up period. Although there is limited literature on the use of splint fiber and ligature wires for pediatric mandibular fractures, the technique should be performed by an experienced surgeon due to its specific application.

Overall, the management of mandibular fractures in pediatric patients requires a comprehensive understanding of the unique challenges and considerations in this population.

## Conclusion

In conclusion, pediatric mandible fractures present unique challenges to maxillofacial surgeons and require careful consideration of various factors. Although the incidence of mandible fractures in children is relatively low compared to adults, understanding their causes, patterns, and locations is crucial for diagnosis and treatment planning. Conservative treatment options, such as closed reduction and immobilization with splints and circumdental arch wiring, should be preferred whenever possible to avoid adverse effects on future skeletal growth and dental development. However, in specific cases, open reduction with internal fixation may be necessary to achieve optimal outcomes. Surgeon providers must tailor their treatment plans by considering the patient’s age, anatomy, and stage of dental development. The use of quartz splint fiber and external fixation with fiber splint can also be effective alternatives to provide occlusal stability without any negative impacts on growth and development. In summary, a multidisciplinary approach is essential for achieving the best outcomes for pediatric patients with mandibular fractures.

## Data Availability

All relevant datasets and their supporting information files generated and/or analyzed during this study are available from the corresponding author upon reasonable request.
