# Correction to: Conservative management of mandibular fractures in pediatric patients during the growing phase with splint fiber and ligature arch wire

**DOI:** 10.1186/s12903-023-03460-7

**Published:** 2023-10-21

**Authors:** Lifeng Li, Kiran Acharya, Bedana Ghimire, Yanqiu Li, Xiaotao Xing, Xiaoru Hou, Lingnan Hou, Xiaoyi Hu

**Affiliations:** 1 Key laboratory of Shaanxi Province for Craniofacial Precision Medicine Research, College of Stomatology, Xi’an Jiaotong University, Xi’an, China; 2 Clinical Research Center of Shaanxi Province for Dental and Maxillofacial Diseases, College of Stomatology, Xi’an Jiaotong University, Xi’an, China; 3 Department of Cranio-Maxillofacial Trauma and Plastic Surgery, College of Stomatology, Xi’an Jiaotong University, Xi’an, China; 4 Shree Birendera Sainik (Army Hospital), Kathmandu, Nepal; 5 Department of Cranio-Maxillofacial Trauma and Plastic Surgery, College of Stomatology, Xi’an Jiaotong University, No 98 Xiwu Road, 710004 Xi’an, Shaanxi People’s Republic of China

**Correction to: BMC Oral Health (2023) 23:601**

10.1186/s12903-023-03309-z

In this article [1], the city and country details for Affiliation 1 was incorrectly published as “Kathmandu Nepal,” instead of “Xi’an, China”.

The original article has been corrected.
